# Fluid Intelligence and Competence Development in Secondary Schooling: No Evidence for a Moderating Role of Conscientiousness

**DOI:** 10.3390/jintelligence10020027

**Published:** 2022-04-28

**Authors:** Naemi D. Brandt, Clemens M. Lechner

**Affiliations:** 1 Department of Psychology, Educational Psychology and Personality Development, von-Melle-Park 5, 20146 Hamburg, Germany; 2 GESIS—Leibniz Institute for the Social Sciences, P.O. Box 12 21 55, 68072 Mannheim, Germany; clemens.lechner@gesis.org

**Keywords:** fluid intelligence, conscientiousness, competence development, secondary school, interaction, NEPS

## Abstract

Fluid intelligence and conscientiousness are important predictors of students’ academic performance and competence gains. Although their individual contributions have been widely acknowledged, less is known about their potential interplay. Do students profit disproportionately from being both smart and conscientious? We addressed this question using longitudinal data from two large student samples of the German National Educational Panel Study. In the first sample, we analyzed reading and mathematics competencies of 3778 fourth graders (*M*_age_ = 9.29, 51% female) and gains therein until grade 7. In the second sample, we analyzed the same competencies in 4942 seventh graders (*M*_age_ = 12.49, 49% female) and gains therein until grade 9. The results of (moderated) latent change score models supported fluid intelligence as the most consistent predictor of competence levels and gains, whereas conscientiousness predicted initial competence levels in mathematics and reading as well as gains in mathematics (but not reading) only in the older sample. There was no evidence for interaction effects between fluid intelligence and conscientiousness. We found only one statistically significant synergistic interaction in the older sample for gains in reading competence, which disappeared when including covariates. Although our findings point to largely independent effects of fluid intelligence and conscientiousness on competence gains, we delineate avenues for future research to illuminate their potential interplay.

## 1. Introduction

Cognitive abilities are a key ingredient for students’ learning progress at school. In particular, fluid intelligence shows substantial and systematic associations with both school grades and different subject-specific academic competencies (Brandt et al. 2020; Deary et al. 2007; Roth et al. 2015). Besides cognitive abilities, inter-individual differences in how people habitually think, feel, and act—their personality traits—explain students’ academic success (Lechner et al. 2017; Poropat 2009, 2014). Here, the Big Five trait conscientiousness emerged as the most consistent predictor of academic performance (Dumfart and Neubauer 2016; Laidra et al. 2007; Lechner et al. 2017) and, to a lesser extent, increases over time in academic performance (Heaven and Ciarrochi 2008; Israel et al. 2019, 2022; Spengler et al. 2016). Whereas previous research was mainly interested in extracting the unique and incremental contributions of cognitive ability and personality, less is known about their possible interaction (but see Ziegler et al. 2012; Lechner et al. 2019). The aim of the current project was to address this lacuna by investigating whether students profit disproportionately from being both smart and diligent: that is, by testing whether interaction effects of fluid intelligence and conscientiousness can add to the explanation of students’ competence gains above and beyond the main effects of these constructs. We addressed this question in two large and representative student samples from the German National Educational Panel Study (NEPS) using moderated latent change score modeling. In the first sample, we analyzed reading and mathematics competencies of 3778 fourth graders and gains therein over three years (until grade 7). In the second sample, we analyzed levels of the same competencies in 4942 seventh graders as well as gains therein over two years (until grade 9).

### 1.1. Fluid Intelligence as the Engine of Learning

The role of cognitive abilities in academic performance at a single point in time but also learning gains is well established. Results from numerous empirical large-scale studies and meta-analyses clearly support the notion that fluid intelligence in particular explains inter-individual differences in students’ competence levels and gains therein over time (Brandt et al. 2020; Deary et al. 2007; Lechner et al. 2019; Roth et al. 2015). Based on the idea that cognitive abilities can be separated into two related but distinct aspects (e.g., Cattell 1943, 1987), researchers have distinguished fluid intelligence (also called g_f_) from crystallized intelligence (also called g_c_), differentiating the process of knowledge acquisition from the resulting knowledge a person has acquired. In other words, whereas fluid intelligence describes how efficiently a person can process novel information, crystallized intelligence captures what the person already knows. Factor analytic and neuronal evidence supports the notion of two distinct cores of cognitive abilities (Lang et al. 2016; Nisbett et al. 2012). Furthermore, developmental research suggests that fluid and crystallized intelligence differ in whether they are shaped by the individual genetic make-up or environmental influences (e.g., Baltes 1987; Baltes et al. 2006). The interplay of these two core features is further formalized in investment theories proposing that increases in competencies (or g_c_) accrue from a continued investment of one’s fluid intelligence (or gf; Ackerman 1996; Cattell 1943, 1987) in a certain subject area, highlighting the role fluid intelligence plays in academic progress.

### 1.2. Does Conscientiousness Amplify the Power of Fluid Intelligence?

Besides the fluid capacities, the investment theory perspective posits that a range of personality traits, which it terms investment traits, determine how people invest their time and effort in intellectual pursuits, thereby contributing to individual differences in competence development across the life span (Ackerman 1996). Investment traits are thought to contribute to learning both in a main effect fashion and by strengthening the association between fluid intelligence and competence development (Lechner et al. 2019; von Stumm et al. 2011; von Stumm and Ackerman 2013; Ziegler et al. 2012). Consistent with the main focus of investment theories, previous research in this tradition has focused primarily on investment traits from the Big Five domain of openness and related constructs such as curiosity and interests (Lechner et al. 2019; Strobel et al. 2019; Zhang and Ziegler 2022). However, we submit that the definition of investment traits as “stable individual differences in the tendency to […] continuously pursue opportunities for effortful cognitive activity” (von Stumm et al. 2011, p. 225) with its emphasis on continuous, effortful engagement is equally applicable to conscientiousness—even though conscientiousness has thus far been largely absent from research inspired by the investment theory tradition.

Conscientiousness encompasses a range of behaviors that, in line with this definition of investment traits, are conducive to learning. These behaviors include active engagement with a set of tasks and materials, sustained effort even in the face of distractions or setbacks, and being diligent and well organized in the learning process (Jackson et al. 2010). Thus, whereas openness may be most conducive to seeking out and exploring new learning opportunities with interest and curiosity (John et al. 2008), conscientiousness may help students invest continuous effort into the learning process and thereby help them translate learning opportunities into actual learning gains. This reasoning is also in line with what is put forth by the invest-and-accrue model of conscientiousness (Hill and Jackson 2016), which assumes that conscientiousness allows students to foster future success by continuously investing efforts in certain tasks and goals.

According to this reasoning, conscientiousness should be helpful in a variety of school-related tasks and affordances, such as completing school work, and therefore support students’ learning progress at school. It is therefore unsurprising that previous evidence from large-scale studies and meta-analyses demonstrated a robust association of conscientiousness with school grades and—to a lesser extent—standardized competence measures (Brandt et al. 2020; Mammadov 2021; Poropat 2009; Spengler et al. 2016). If conscientiousness acts as an investment trait, conscientious students can furthermore invest their cognitive abilities more efficiently and continuously in goals that foster their future learning gains compared to students with lower levels of conscientiousness. That is, the strongest increases in competencies should result from both an efficient processing of newly learned information, which is facilitated by high fluid intelligence, and a continuous disciplined repetition or application of the newly learned information in different contexts, which is facilitated by high conscientiousness. Statistically, this would imply a “synergistic” interaction in which both fluid intelligence and conscientiousness are necessary and non-sufficient conditions for learning gains, such that the highest rate of learning is observed among students who score high on both traits.

### 1.3. Does Conscientiousness Compensate for low Levels of Fluid Intelligence?

In view of the negative correlation between cognitive abilities and conscientiousness reported in some adolescent samples (Lechner et al. 2017; Moutafi et al. 2004; but see also Allik et al. 2004; Murray et al. 2014), another perspective holds that gains in academic competencies can be achieved either with high levels of cognitive abilities or high levels of conscientiousness. That is, conscientiousness might compensate for lower levels of cognitive abilities, indicating a “compensatory” interaction. Fluid intelligence and conscientiousness, from this perspective, are both sufficient but non-necessary conditions for competency gains. This means that even students with low levels of cognitive abilities can achieve competency gains so long as these students compensate for their low fluid intelligence levels with high levels of conscientiousness. In turn, higher fluid intelligence might compensate the disadvantages in terms of learning gains that normally arise from a lack of conscientiousness. Some have argued that lower levels of fluid intelligence should increase students’ efforts to act conscientiously, particularly in competitive environments, to keep up with more intelligent classmates and higher academic demands (Moutafi et al. 2004). In our view, compensatory interactions between fluid intelligence and conscientiousness appear less plausible than synergistic interactions because fluid intelligence and conscientiousness are more likely to be necessary, rather than non-necessary, conditions for learning gains.

### 1.4. Previous Evidence on Interactions

Only few studies have tested possible interactions between cognitive abilities and conscientiousness, and their findings are mixed. Bergold and Steinmayr (2018) found a positive interaction effect between conscientiousness and reasoning on grades in high school students, indicating that associations between reasoning and grades were stronger when conscientiousness was higher (i.e., there was a synergistic interaction). Likewise, Sorjonen et al. (2021) found evidence for a synergistic interaction effect between conscientiousness and intelligence measures in young adulthood on earlier reported high school GPA. Conversely, Heaven and Ciarrochi (2012) did not find any interaction but only main effects on grades. In college student samples, interaction effects occurred more consistently than in younger samples, suggesting that students’ performance in academically demanding environments benefits from students’ being both highly able and conscientious (Beaujean et al. 2011; Di Domenico and Fournier 2015; Ziegler et al. 2009). In young adults in vocational training, however, no statistically significant interaction between self-control (a facet of conscientiousness) and fluid intelligence in predicting grades was found (Schmidt et al. 2020).

Two key limitations shared by most prior studies concern the outcome measures and the research designs they used. Specifically, no previous study investigated the role of interaction effects between fluid intelligence and conscientiousness for academic competence gains in students as measured with objective competence tests. Most studies instead focused on school grades only, which are only moderately related to students’ actual competencies as measured with standardized achievement tests (e.g., Lechner et al. 2017; Borghans et al. 2016). Moreover, most studies used cross-sectional designs, rendering these studies unable to properly assess learning gains in the form of changes over time in competencies.

### 1.5. The Present Study

With the current study, we aim to provide new knowledge about the interplay between fluid intelligence and conscientiousness in explaining learning gains over time in two key academic competence domains, reading and mathematics.

Expanding investment theory’s focus on traits from the domain of openness (e.g., Ackerman 1996; von Stumm and Ackerman 2013; Ziegler et al. 2012), we examined whether fluid intelligence interacts with conscientiousness in predicting students’ academic competencies and gains therein over a two- to three-year period. We used longitudinal data of two large representative student samples from NEPS Starting Cohorts 2 and 3.

To start with, we investigated the single roles of fluid intelligence and conscientiousness in competence levels at a certain time (i.e., in grade 4 and grade 7) and in competence gains over time. In the next step, we examined whether conscientiousness works as an investment trait and thus might leverage students’ ability to translate their fluid intelligence into (gains in) academic competencies. We expected that this interaction should be related to both immediate competence but also to competence gains across two and three years of schooling. As competence measures, we used competencies in two key domains, reading (in German) and mathematics assessed with standardized achievement tests within the NEPS. We tested the following hypotheses that we preregistered (along with the analytic plan) on the Open Science Foundation (OSF) before conducting any data screening or data analyses (see https://osf.io/prdf7, accessed on 25 November 2021).

**Hypothesis** **1.** *Fluid intelligence will predict higher baseline levels and stronger increases in math and reading competencies.*

**Hypothesis** **2.** *Conscientiousness will predict higher baseline levels and stronger increases in math and reading competencies.*

**Hypothesis** **3.** *Fluid intelligence and conscientiousness will have a positive synergistic interaction effect on baseline levels in math and reading competencies.*

**Hypothesis** **4.** *Fluid intelligence and conscientiousness will have a positive synergistic interaction effect on increases in math and reading competencies.*

## 2. Method

In the current study, we analyzed publicly available secondary data from the German National Educational Panel Study (NEPS) that can be downloaded as anonymized scientific use files from the NEPS website after concluding a data use agreement with the Leibniz Institute for Educational Trajectories (https://www.neps-data.de/Data-Center/Data-Access). All data collections that took place as part of NEPS were reviewed and approved under German law and research ethics codes. Written informed consent to participate in this study was provided by the participants’ legal guardian/next of kin.

### 2.1. Participants

Data came from two longitudinal student samples from the NEPS, an ongoing large-scale, multi-cohort study on educational trajectories and skill development (for a detailed description, see Blossfeld et al. 2011). We used data from Starting Cohort 2 (SC2) and Starting Cohort 3 (SC3). Starting Cohort 2 comprises a representative sample of 2949 four-year-old children first assessed in kindergarten. We took data from waves 6 and 9 when children were in grade 4 (elementary school) and grade 7 (secondary school) assessed in 2015/16 and 2018/19, respectively. Starting Cohort 3 comprises a representative sample of originally 5778 students first assessed at grade 5, the first year of secondary schooling. We used data of waves 3 and 5, when children were in grade 7 and grade 9, collected in 2012/13 and 2014/15, respectively. The NEPS data documentation describes the original sample size of SC2 with n = 6954 at grade 4 and SC3 with n = 6838 at grade 7. We only included those students that provided data on standardized achievement tests in both waves and who attended regular schools such as academic tracks (Gymnasium), intermediate tracks (Realschule), vocational tracks (Hauptschule), or comprehensive schools (Gesamtschule) in both samples.

When applying these inclusion and exclusion criteria, SC2 comprised *n* = 3778 fourth graders at T1, with 51% being female, reporting an average age of 9.29 years (*SD* = .47). At T2 in grade 7, 84% of the students were attending an academic track (or a respective stream in a comprehensive school). SC3 comprised *n* = 4942 seventh graders at T1, with 49% being female, with an average age of 12.49 years (*SD* = .61). A total of 50% of the students were attending an academic track (or a respective stream in a comprehensive school) in grade 7. As a robustness check, we repeated our analyses including all students that provided data at either T1, T2, or at both waves (please see Tables S1–S7 in the online Supplementary Materials). The pattern of the results remained highly stable.

### 2.2. Measures

#### 2.2.1. Fluid Intelligence (Reasoning)

In SC2 (grade 2) and SC3 (grade 5), students took a 12-item matrices test (NEPS-MAT). Matrices tests are considered the best single indicator for reasoning and a good indicator for fluid intelligence or g_f_ (Nisbett et al. 2012; Gignac 2015). NEPS-MAT is similar to Raven’s Standard Progressive Matrices and was developed and validated specifically for NEPS (for further information, see Haberkorn et al. 2012). Each test item is binary scored as correct (1) or incorrect (2). We used sum scores of these items in three sets with 4 binary items each as manifest indicators for a latent variable. The internal reliability of NEPS-MAT in terms of the reliability index ω (McDonald 1999) was satisfactory in both samples, with ω = .80 in SC2 and a reasonable ω = .69 in SC3. In a previous validation study (Lang et al. 2014), internal consistencies (Cronbach’s alpha) were .60 in fifth grade and .71 in ninth grade. Nevertheless, we used measurement error-free latent variables in our analyses that correct for the unreliability of indicators. Thus, the (un-)reliability of the observed scores was not of major importance to our study.

#### 2.2.2. Conscientiousness

In both starting cohorts, conscientiousness was assessed with short scales. In SC2, we used four items asking students to indicate whether they “handle their working materials carefully”, whether they “complete all tasks with great care”, whether “they give up quickly when something is difficult” (reversed item), and whether “they try hard, even when tasks are difficult” on a 4-point scale ranging from 1 (*does not apply at all*) to 4 (*fully applies*). These items were assessed between 10/2015 and 01/2016 in grade 4 and 10/2018 and 04/2019 in grade 7. Model-based reliability was reasonable with ω = .69.

In SC3, students were asked to rate two items of the widely used Big Five Inventory-10 (BFI-10; Rammstedt 2007). The BFI-10 measures conscientiousness with two items, one of which is reverse-keyed to control for acquiescent responding. These were “I am easy-going and tend to be a bit lazy (reversed item)” and “I am thorough”. Students rated the two items on a five-point scale ranging from 1 (*does not apply at all*) to 5 (*fully applies*). These items were assessed between 10/2012 and 01/2013 in grade 7 and 10/2014 and 01/2015 in grade 9. As is typical for (ultra-)short scales, split-half reliability of the manifest scale was relatively low with *r* = .53 in the current study. As often occurs for short scales, test–retest reliability is higher than internal consistency; for example, Rammstedt and John (2007) reported a retest stability of *r* = .83 in German samples. Despite their lower reliability, prior research shows that personality short scales perform at a similar, and in some cases even higher, level to (much) longer scales as regards predictive validity (e.g., Thalmayer et al. 2011; Rammstedt et al. 2021). Moreover, as for fluid intelligence, we used latent variables for conscientiousness that account for the unreliability of the observed items.

#### 2.2.3. Academic Competencies

Within the NEPS, students took standardized achievement tests, including a test assessing reading competencies in German and a test assessing mathematical competencies (SC2, grades 4 and 7; SC3 grades 7 and 9). Tests were administered individually in a paper-pencil mode at school or at home and were limited to approximately 30 min. Tests were presented in different versions based on previous competence levels as easy and difficult versions (grade 7) or as easy, intermediate, and difficult versions (grade 9). The test for reading skills in German comprised 31 items in grade 4 (SC2), 42 items in grade 7 (SC2 and SC3), and 30 (easy version) or 32 (difficult version) items in grade 9 (SC3) on finding information in the text, drawing text-related conclusions, and reflecting and assessing presented as single multiple-choice, complex multiple-choice, and matching items. The reliabilities of the test were reported as good (WLE reliability = .75–97; Scharl et al. 2017, 2021).

The mathematical skill test comprised 24 items in grade 4 (SC2), 21 items in grade 7 (SC2 and SC3), and 34 items in grade 9 (SC3) on quantity, space and shape, change and relationships, and data and change presented as single multiple-choice, complex multiple-choice, and short constructed response items. The reliabilities of the test were reported as good (WLE reliability = .73–81; Kock et al. 2021; Schnittjer et al. 2020; van de Ham et al. 2018).

For all tests, we used weighted likelihood estimates (WLEs) from item response theory (IRT) models provided by the NEPS. For our longitudinal analyses of competency gains, we used the “uncorrected” WLE scores that are statistically linked across waves based on item difficulty parameters. This allows for competence comparisons of adjacent waves and analyses of change across secondary schooling. More information on the tests and scaling procedures is provided by Rohm et al. (2017), Scharl et al. (2017), and Scharl et al. (2021), for reading skills in German, and by Kock et al. (2021), Schnittjer et al. (2020), and van de Ham et al. (2018), for mathematical skills.

#### 2.2.4. Covariates

In all our analyses, we controlled for the influence of a set of variables that prior research (e.g., Lechner et al. 2021) has shown to be linked to both our predictors (i.e., fluid intelligence and conscientiousness) and outcomes (i.e., academic competencies), suggesting that these variables might act as confounders. These variables were participants’ gender (coded as 0 = male, 1 = female) and parental highest occupational prestige (HISEI; Ganzeboom 2010) with a possible value range between 11.56 and 88.96. For gender and HISEI, we used harmonized information from all assessment waves of the respective starting cohorts. We furthermore controlled our analyses for the variable “school track” students were attending in grade 7 (SC2 and SC3). This variable was coded as a dummy variable with 0 = academic track and 1 = non-academic track.

### 2.3. Analytical Strategy

**Figure 1:**
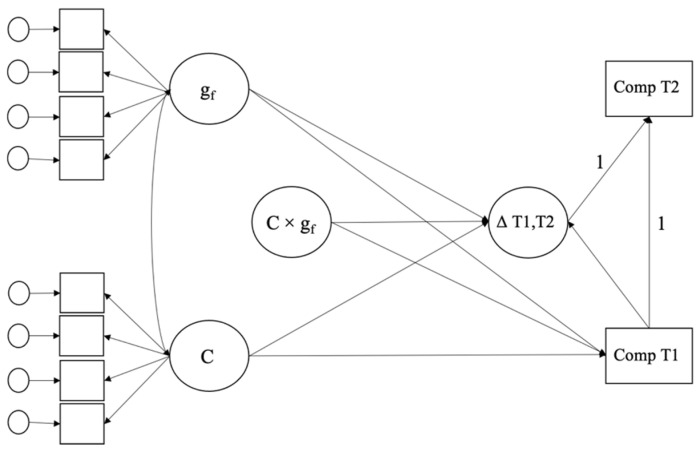
Schematic representation of the latent change regression model predicting initial levels (Comp T1) and changes (∆ T1,T2) in competencies from T1 to T2 from conscientiousness (C), fluid intelligence (g_f_), and their interaction (C × g_f_). *Note.* Squared boxes represent manifest indicators, whereas bubbles represent latent variables. For parsimony, covariates are omitted.

To capture changes in reading and mathematical competence, we computed latent difference scores (also called change regression models, e.g., McArdle 2009) between the WLE estimates of the two waves in each sample (grades 4 and 7 SC2 as well as grades 7 and 9 in SC3). We modeled conscientiousness and fluid intelligence as continuous latent variables (see Figure 1 for a schematic representation of the model). Measurement models of the latent variables were identified using the effect coding method (Little et al. 2006). We then fitted a series of regression models including fluid intelligence and conscientiousness as latent variables predicting baseline competence and change scores of academic competencies to investigate Hypotheses 1 and 2. We specified models for reading and mathematics competence separately.

To investigate Hypotheses 3 and 4, we used moderated structural equation modeling (Klein and Moosbrugger 2000) to specify latent interactions of fluid intelligence and conscientiousness. We used this interaction term as a predictor of the baseline competence in grades 4 (SC2) and 7 (SC3) as well as a predictor of change scores (∆grade4–grade7 in SC2 and ∆grade7–grade9 in SC3) of academic competencies in reading and mathematics. We centered latent means of fluid intelligence and conscientiousness in interaction analyses by restricting their means to zero.

In all our analyses, we used the robust maximum likelihood estimator and included all available data by using the full information maximum likelihood algorithm. We considered the nested data structure (students nested in classes) by using the type = complex option in Mplus based on students’ classID. In the interaction models, however, type = random needs to be specified. We used established fit criteria for evaluating the goodness of fit of our models that included the main effects only (CFI > .90–.95; RMSEA < .08–.05, SRMR < .11–.08; Hu and Bentler 1999; Schermelleh-Engel et al. 2003). In models with latent interaction terms, no overall model fit in terms of CFI, RMSEA, or SRMR can be estimated (Klein and Moosbrugger 2000). Therefore, we computed ∆χ^2^ values based on the log-likelihood values and scaling correction factors (see Hildebrandt et al. 2009). Because of the large sample size and multiple testing, we chose a stricter alpha level of *p* < .05 and only interpreted findings being significant at the *p* < .01 level. We report effect sizes following the guidelines of Funder and Ozer (2019). The main analyses were conducted in Mplus (8.5; Muthén and Muthén 2020) by using the MplusAutomation package (0.7–3; Hallquist and Wiley 2018) in R (4.0.2; R Core Team 2020). Model codes can be found on the project’s OSF site (https://osf.io/75gah/ accessed on 25 November 2021).

## 3. Results

Table 1 and Table 2 show means, standard deviations, and correlations of manifest fluid intelligence and conscientiousness scale scores, WLE estimates of academic competencies in reading and math, and covariates. Students, on average, gained 0.51 scale points in reading (Cohen’s *d* = .39) in SC2 and 0.46 scale points in SC3 (Cohen’s *d* = .37). In mathematics, the average student gained 1.01 scale points (Cohen’s *d* = .87) in SC2 and 0.74 scale points in SC3 (Cohen’s *d* = .61). The standard deviations, however, suggest considerable inter-individual differences in competence gains in the two- and three-year intervals.

In both samples, fluid intelligence showed statistically significant associations with later assessed competencies in reading and mathematics, with small effect sizes in SC2 and medium effect sizes in SC3. Conscientiousness was positively related to reading competencies in both samples with small effect sizes, but only very small effect sizes for mathematic competencies in SC2. Whereas fluid intelligence and conscientiousness showed a small positive association in SC2, they were not statistically significantly associated in SC3. Overall, non-academic track students scored lower in fluid intelligence and reported lower levels of conscientiousness. In contrast, students with parents having more prestigious jobs scored higher in fluid intelligence but reported lower conscientiousness. In both samples, females were found to be slightly more conscientious than males, whereas differences between boys and girls in fluid intelligence were inconsistent across samples, with girls scoring higher in SC2 but not in SC3. In the following, we first report the results of our latent change score models that included fluid intelligence, conscientiousness, and the interaction thereof (unconditional models). In the second step, we report the models that additionally included the covariates school type, gender, and parental occupational prestige (conditional models).

### 3.1. Predicting Reading Competence

**Unconditional models**. The model fits of our models including the variables of main interest in this study were acceptable in SC2 and good in SC3 (see Table 3 and Table 4 for model fits and parameter estimates). Across both samples, fluid intelligence was most consistently related to reading baseline levels with large effect sizes (grade 4 in SC2 and grade 7 in SC3). Furthermore, fluid intelligence predicted competence gains in reading from grades 4 to 7 (SC2) and from grades 7 to 9 (SC3) with medium effect sizes. That is, in line with Hypothesis 1, students who scored higher in fluid intelligence showed both higher baseline levels and stronger increases in reading competencies across grades. Only partly in line with Hypothesis 2, conscientiousness was associated with baseline levels of reading competence in grade 7 (SC3) with a small effect size but not in grade 4 (SC2) and did not predict reading increases in both samples.

**Figure 2:**
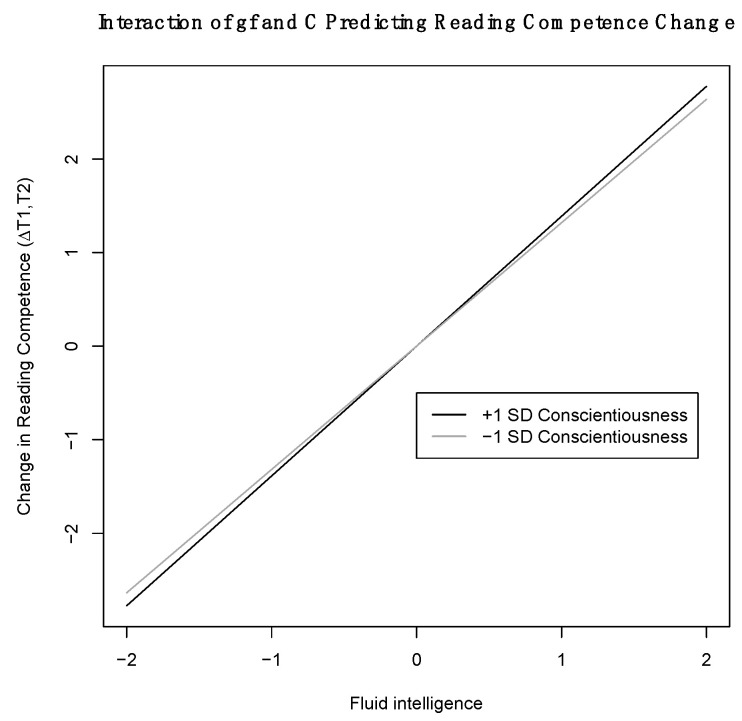
Interaction between fluid intelligence and conscientiousness in predicting reading competence change in SC3. *Note.* The dark gray line represents the association between fluid intelligence and change in reading competence for high levels of conscientiousness (+1 SD above the sample mean), whereas the light gray line represents the same association for low levels of conscientiousness (–1 SD below the sample mean).

There was only one statistically significant interaction effect: In line with Hypothesis 4, scoring higher in fluid intelligence and reporting higher scores in conscientiousness resulted in stronger gains in reading competence across time in SC3 (see Figure 2^1^ and Table 4; estimates for the simple slopes were 0.36 for −1 SD in conscientiousness and 0.43 for +1 SD in conscientiousness). The effect size of the interaction effect (standardized regression coefficient of .05) was very small and considerably smaller than the main effect of fluid intelligence (B = .20), whereas the main effect of conscientiousness was statistically non-significant. The interaction effect accounted for an additional explanation of 0.1% of the variance in reading competencies. When comparing the model fits of both models, Table 5 indicates that the model with the interaction effect fitted the data worse compared to the model without the interaction effect. Furthermore, AIC and aBIC were slightly higher in models with interaction effects. We found no evidence for an interaction effect with regard to the baseline levels of reading competence in SC3, and no interactions at all in SC2. Thus, the results do not provide much support for our hypotheses that fluid intelligence and conscientiousness may interact in predicting (gains in) reading competencies.

**Conditional models.** When including the covariates school type, gender, and parental occupational prestige, the specified models including baseline levels and changes in reading competencies still showed an acceptable (SC2) or good (SC3) fit with the data (see Table 3 and Table 4). In terms of fluid intelligence, the results remained largely unchanged. Whereas estimates reduced a little bit in size, the overall pattern was supported in that fluid intelligence was associated with both the baseline level and competence increases across both samples. Effect sizes remained large in the conditional models. In stark contrast, we found no statistically significant association of conscientiousness with reading competence in both samples when including the covariates. Additionally, the interaction effect between fluid intelligence and conscientiousness was no longer statistically significant. Across both samples, school track and parental occupational prestige were statistically significantly related to baseline levels (medium effect sizes) and predicted gains in reading competence in both samples (small effect sizes). Gender was also related to baseline levels and change in reading in SC3, with girls showing higher baseline levels in grade 7 and stronger increases in reading from grade 7 to grade 9 than boys (small effect size). Follow-up exploratory multi-group analyses pointed to a very small interaction effect in girls visiting academic school tracks in SC3 (*B* = .09, *p* = .015) that, however, was not statistically significant at an alpha level of α = 0.01. That is, girls, but not boys, on academic school tracks had higher reading scores when having higher scores in both fluid intelligence and conscientiousness compared to girls with lower levels of conscientiousness.

In models with covariates, the explained variance increased from 23.5/25.1% to 27/28% in SC2 and from 39.6/39.7% to 42.5/42.5% in SC3 in the baseline and interaction models, respectively.

### 3.2. Predicting Mathematic Competence

**Unconditional models**. The model fits of our models including fluid intelligence, conscientiousness, and mathematic competencies were acceptable in SC2 and good in SC3 (see Table 6 and Table 7 for model fits and parameter estimates). Very much in line with what we found for reading competence, fluid intelligence was most consistently related to mathematic baseline levels (grade 4 in SC2 and grade 7 in SC3) and furthermore predicted competence gains in mathematics from grades 4 to 7 (SC2) and from grades 7 to 9 (SC3) with large effect sizes. That is, in line with Hypothesis 1, students who scored higher in fluid intelligence showed both higher baseline levels and stronger increases in mathematic competencies across grades. Additionally, in line with the results for reading competence, conscientiousness was neither associated with mathematic competence baseline levels nor changes in SC2. However, conscientiousness was associated with mathematic competencies at baseline in grade 7 (SC3) and predicted change in mathematic competencies from grade 7 to grade 9 (SC3) with small effect sizes. Thus, Hypothesis 2 can be confirmed in higher grades in SC3 but not in SC2. In both samples, we found no evidence for an interaction effect of fluid intelligence and conscientiousness on mathematic competence.

**Conditional models**. When including the covariates, model fits remained acceptable (SC2) or good (SC3, see Table 6 and Table 7 for model fits and parameter estimates). For SC2, the pattern of results remained fully stable with statistically significant and large effects of fluid intelligence on both baseline levels and competence gains in mathematics. For SC3, however, greater changes occurred in terms of conscientiousness. When covariates entered the model, conscientiousness was not a statistically significant predictor of baseline levels or changes in mathematic competencies anymore. At the same time, the amount of explained variance increased from 12.9/14% to 17.6/18.9% in SC2 and from 19.6/20% to 24.6/24.7% in SC3 in the baseline and interaction models, respectively.

## 4. Discussion

Research has identified students’ cognitive abilities and personality traits—especially conscientiousness—as important predictors of learning gains in school (Heaven and Ciarrochi 2008; Israel et al. 2019, 2022; Spengler et al. 2016). However, comparatively little is known about whether students profit disproportionately from being both smart and diligent. In two large student samples from NEPS, we therefore investigated interactive effects between fluid intelligence and conscientiousness to predict students’ baseline competence levels and competence gains over a two- to three-year period in two domains—reading and mathematics.

Three main findings stand out: First, reasoning (assessed with a matrices test) as an indicator of fluid intelligence was consistently associated with competence levels in reading and mathematics and predicted competence gains therein across both samples. The effect of fluid intelligence remained completely stable even when controlling for possible confounders such as parental occupational prestige, academic track, and gender. In line with previous research, fluid intelligence varied in its relevance for competencies in different school subjects, exhibiting stronger associations with math than with reading test scores (Brandt et al. 2020; Meyer et al. 2019). Second, conscientiousness was associated with competence levels in reading and mathematics in grades 4 and 7 and also predicted competence gains in mathematics from grade 7 to grade 9. As in previous work (e.g., Brandt et al. 2020), effect sizes were very small. In stark contrast to reasoning, however, none of these associations remained statistically significant when considering the covariates. Third, and most importantly, evidence for interaction effects between fluid intelligence and conscientiousness was absent in SC2 and weak in SC3 with their different measures of conscientiousness. We found only one statistically significant interaction effect when predicting reading gains in the older sample from grade 7 to grade 9. Again, this interaction effect was very small and disappeared when covariates were included. These findings underscore the prominent role cognitive abilities, and fluid intelligence in particular, play in competence levels and gains. By contrast, conscientiousness showed rather small independent main effects, demonstrating the need to better understand why non-cognitive characteristics such as conscientiousness show inconsistent associations with students’ competencies especially when being assessed with standardized competence tests.

In line with propositions from investment theories (e.g., Ackerman 1996; Cattell 1943, 1987) and previous empirical evidence (e.g., Deary et al. 2007; Lechner et al. 2019), levels and gains in competencies result as a consequence of a continued investment of a student’s fluid intelligence into a specific subject area. As fluid intelligence enables people to solve complex problems and new demands using different aspects of reasoning, strong associations with standardized competence tests are expectable and well documented (Brandt et al. 2020; Israel et al. 2019; Meyer et al. 2019). Our findings support these findings and expand them by showing that fluid intelligence not only predicts competence levels but also predicts competence gains over time at various stages of secondary schooling.

Investment theories further suggest that specific personality characteristics modify how people invest their time and effort in intellectual pursuits and thereby contribute to individual difference in competence gains (Ackerman 1996). Whereas previous research supported this notion, for instance, in terms of openness and interest (e.g., Lechner et al. 2019; Strobel et al. 2019), we made the claim that conscientiousness is also important by helping students to invest continuous effort into the learning process and thereby convert their cognitive potential into actual competence gains. The support for this assumption in our study was, however, weak. We found one very small interaction effect only between fluid intelligence and conscientiousness predicting reading gains from grade 7 to grade 9. Although this effect pointed in the direction that conscientiousness can amplify positive effects of fluid intelligence on competence gains, it disappeared when considering gender, school track, and parents’ occupational prestige differences. In the following, we discuss four possible explanations for why conscientiousness and fluid intelligence did not interact systematically in our study.

First, we assessed competence gains with standardized achievement tests and not school grades. Meta-analyses found strong associations between conscientiousness and grades (Mammadov 2021; Poropat 2009), and some previous research found synergistic interactions between conscientiousness and fluid intelligence in predicting school grades in eleventh graders (Bergold and Steinmayr 2018). However, associations between conscientiousness and standardized achievement tests are often clearly weaker than those with school grades (e.g., Brandt et al. 2020). In this regard, it is important to realize that school grades and standardized achievement tests are not mutually interchangeable indicators of achievement. Instead, they are only weakly related and reflect partly distinct influences (e.g., Borghans et al. 2016; Hübner et al. 2022; Lechner et al. 2017). Even though standardized competence tests, as used in NEPS and similar educational studies, do assess curriculum-based content similar to what is required from students in class, not all of this content is explicitly taught in class. In turn, not everything that is taught in class and reflected in grades is assessed in standardized competence tests (Brookhart et al. 2016; Willingham et al. 2002). Grades reflect how well a student worked during the school year and learned the material presented in class, so that continuous learning efforts pay off. It appears that previous continuous learning efforts cannot be generalized to less familiar testing situations where students are confronted with slightly different materials and content as with standardized achievement tests. The limited evidence for interaction effects indicates that this is also true at higher levels of fluid intelligence.

At the same time, in contrast to grades, standardized achievement tests within the NEPS are without consequences for students’ educational careers, reducing the motivation for persistent test preparation. Future studies should, thus, study interaction effects between fluid intelligence and conscientiousness using high-stakes assessments such as standardized learning level assessments at the end of middle school or college admission tests.

Second, fluid intelligence and students’ academic competencies were assessed using objective tests, whereas conscientiousness was measured via students’ self-reports on two different short scales. Although self-reports provide important insights into the inner states of a person (e.g., Vazire 2010), other reports can add complementary information particularly with regard to the behavioral aspects of personality such as conscientiousness (e.g., Brandt et al. 2021). Because the students rated their conscientiousness in the classroom in the NEPS assessments used in our study, it is reasonable to assume that the students evaluated their conscientiousness with the conscientiousness of classmates in mind. Students very high in conscientiousness surrounded by highly conscientious peers might underestimate their conscientiousness, whereas those low in conscientiousness might overestimate their score in a context of rather unconscientious peers. Further analyses should therefore try to disentangle reference group effects in self-ratings that might attenuate the association of conscientiousness with standardized achievement test scores.

Third, the interaction effect emerged only in the older sample covering competence gains from grade 7 to grade 9 in SC3. This might indicate that synergistic effects of conscientiousness are more likely in more demanding learning environments. In college student samples, interaction effects occurred more consistently than in younger samples, suggesting that students benefit from being both highly able and conscientious (Beaujean et al. 2011; Di Domenico and Fournier 2015; Ziegler et al. 2009). In young adults in vocational training, however, no statistically significant interaction between self-control (a facet of conscientiousness) and fluid intelligence was found (Schmidt et al. 2020). Comparing school and college settings, they differ strongly in how structured they are. In less structured environments, expectations on behavior are less clear, giving individual differences more room to become more visible and have more potential to create differences in academic performance (e.g., Barrick 2005).

Fourth, although personality traits are relatively stable constructs across time, they do change particularly through adolescence and young adulthood (Mõttus et al. 2019; Roberts and DelVecchio 2000). Although traits such as conscientiousness are assumed to mature when students get older, previous research pointed to temporal dips in these maturational patterns in early adolescence (e.g., Luan et al. 2017; Van den Akker et al. 2014). That is, students who are more conscientious at baseline are potentially less conscientious at T2. Such decreases in conscientiousness potentially diminish associations with competency gains.

### Limitations and Outlook

Besides the strength of this study in using two large, longitudinal datasets from the NEPS to study competence gains with standardized competence assessments, some limitations call for further research. First, we used short (SC2) and ultra-short (SC3) assessments of conscientiousness in both samples. Although ultra-short measures of conscientiousness have shown comparable criterion validity to longer measures, for instance, in terms of associations with academic performance indicators (e.g., Poropat 2009; Rammstedt et al. 2021; Thalmayer et al. 2011), they usually cannot capture the full breadth of the construct. By contrast, the narrower construct of reasoning was measured with 12 items, resulting in a better construct coverage and higher reliability. Despite reasoning being a strong indicator of fluid intelligence (e.g., Nisbett et al. 2012), it also cannot capture the full conceptual breadth of the construct. Short measures may (over)represent specific facets of the global trait and do not allow for facet-level analyses. The latter point is important because previous research suggested that different facets differ in their predictive validity for academic performance (e.g., Corker et al. 2012; Kretzschmar et al. 2016; Noftle and Robins 2007). In high school, particularly aspects of conscientiousness that tap into aspects of achievement striving and diligence are more related to academic performance than a student’s orderliness as per Bergold and Steinmayr’s (2018) study. This was also true for interaction effects: Although both the two-item short scale and the four-item short scale fielded in NEPS SC3 and SC2, respectively, do cover the industriousness/productiveness facet of conscientiousness, they also contain content related to orderliness/organization. The short nature of the scale meant that we could not test for potential differential effects of these conscientiousness facets (see, e.g., Rammstedt et al. 2018, in this journal). Relatedly, Ziegler et al. (2009) found facet-specific synergistic interaction effects in low- and high-performing college students. Achievement striving interacted with cognitive abilities in the low-performing group only. The authors explain this finding with the different motivational and behavioral pattern underlying different facets of conscientiousness. Future studies should investigate interaction effects of fluid intelligence and conscientiousness in competencies in standardized tests using longer inventories and test whether facet-specific interaction effects can be found.

We used standardized achievement tests to assess students’ competencies free of teachers’ subjective evaluations. These tests are developed especially for use within the NEPS, based on students’ curriculum, and evaluated in terms of their quality, for instance, regarding test fairness, item difficulties, and reliability. Although tests were reported as fair and reliable, the authors claim that not all competence levels were captured equally well (Kock et al. 2021; Scharl et al. 2017, 2021). In particular, whereas low performance in reading and mathematical skills was accurately measured, tests were less precise in assessing high-performance students. Potentially, conscientiousness amplifies the effect of fluid intelligence on competence gains only at high-performance levels as interaction effects were found more consistently in college student samples than in younger samples (Beaujean et al. 2011; Di Domenico and Fournier 2015; Ziegler et al. 2009). Future studies should test this.

Finally, although we used longitudinal data and controlled for important covariates, our study design is correlational in nature, prohibiting causal inferences. At the same time, the timing when constructs were assessed within the NEPS differed somehow between constructs, limiting the comparability of effect sizes between constructs. Future studies should replicate findings using same-distanced measures.

## 5. Conclusions

Do students profit disproportionately from being both smart and diligent? Evidence from two large student samples from NEPS particularly highlights the independent role of fluid intelligence (more specifically, reasoning as measured through a matrices test) in competence levels in reading and mathematics as well as gains therein. Different from its well-established effects on school grades, conscientiousness had only small and less consistent main effects on students’ baseline competence levels and competence gains. Moreover, contrary to our hypotheses based on the investment theory perspective, there was no evidence for interaction effects between fluid intelligence and conscientiousness. Our results call for further research on the specific circumstances under which conscientiousness does or does not contribute to student learning, including through possible intelligence–conscientiousness interactions.

## Figures and Tables

**Table 1:** Means, standard deviations, and correlations of SC2.

Variable	*M*	*SD*	1	2	3	4	5	6	7	8
1. Fluid intelligence	5.53	3.65								
2. Conscientiousness	3.26	0.51	.04							
			[.01, .07]							
3. Female	0.51		.05 *	.04						
			[.02, .08]	[.00, .07]						
4. HISEI	64.69	17.56	.06 *	.04	−.01					
			[.03, .09]	[.01, .07]	[−.04, .02]					
5. Non-academic track	0.16		−.07 *	−.13 *	−.00	−.20 *				
			[−.10, −.03]	[−.16, −.09]	[−.04, .03]	[−.23, −.16]				
6. Reading WLE grade 4	−0.29	1.29	.13 *	.09 *	.06 *	.27 *	−.26 *			
			[.10, .17]	[.06, .12]	[.03, .10]	[.24, .30]	[−.29, −.22]			
7. Reading WLE grade 7	0.21	1.27	.10 *	.08 *	.08 *	.30 *	−.23 *	.57 *		
			[.06, .14]	[.04, .12]	[.04, .12]	[.26, .33]	[−.27, −.19]	[.54, .59]		
8. Math WLE grade 4	4.87	1.10	.19 *	.11 *	−.06 *	.30 *	−.26 *	.62 *	.49 *	
			[.16, .22]	[.07, .14]	[−.10, −.03]	[.27, .33]	[−.29, −.23]	[.60, .64]	[.46, .53]	
9. Math WLE grade 7	5.88	1.21	.16 *	.14 *	−.14 *	.31 *	−.27 *	.52 *	.57 *	.65 *
			[.12, .20]	[.10, .18]	[−.18, −.11]	[.27, .34]	[−.31, −.23]	[.50, .55]	[.53, .62]	[.62, .67]
*Note. M* and *SD* are used to represent mean and standard deviation, respectively. For the binary variables gender and school track, proportions are shown. Values in square brackets indicate the 95% confidence interval for each correlation. * indicates *p* < .01.										

**Table 2:** Means, standard deviations, and correlations of SC3.

Variable	*M*	*SD*	1	2	3	4	5	6	7	8
1. Fluid intelligence	4.53	4.00								
2. Conscientiousness	3.25	0.85	.01							
			[−.02, .04]							
3. Female	0.49		−.01	.18 *						
			[−.04, .01]	[.16, .21]						
4. HISEI	57.26	19.74	.14 *	.05 *	.01					
			[.11, .17]	[.02, .08]	[−.02, .05]					
5. Non-academic track	0.50		−.24 *	−.05 *	−.06 *	−.39 *				
			[−.26, −.21]	[−.08, −.02]	[−.09, −.03]	[−.42, −.36]				
6. Reading WLE grade 7	0.87	1.36	.15 *	.04 *	.10 *	.31 *	−.45 *			
			[.12, .17]	[.01, .07]	[.08, .13]	[.27, .34]	[−.48, −.43]			
7. Reading WLE grade 9	1.33	1.12	.20 *	.03	.10 *	.33 *	−.44 *	.63 *		
			[.17, .23]	[.00, .06]	[.07, .13]	[.29, .36]	[−.47, −.42]	[.62, .65]		
8. Math WLE grade 7	0.87	1.22	.24 *	−.01	−.14 *	.35 *	−.51 *	.60 *	.52 *	
			[.21, .26]	[−.04, .02]	[−.17, −.11]	[.32, .38]	[−.53, −.49]	[.59, .62]	[.50, .54]	
9. Math WLE grade 9	1.61	1.20	.26 *	.01	−.12 *	.37 *	−.51 *	.57 *	.58 *	.74 *
			[.24, .29]	[−.02, .04]	[−.15, −.09]	[.34, .40]	[−.53, −.49]	[.55, .59]	[.56, .60]	[.72, .75]
*Note. M* and *SD* are used to represent mean and standard deviation, respectively. For the binary variables gender and school track, proportions are shown. Values in square brackets indicate the 95% confidence interval for each correlation. * *p* < .01.										

**Table 3:** Latent change score models predicting reading competence baseline levels and gains in SC2.

	Model 1: Baseline									Model 2: Interaction							
	Competence T1					Change				Competence T1				Change			
Predictor	Est	*p*	99% CI			Est	*p*	99% CI		Est	*p*	99% CI		Est	*p*	99% CI	
*Unconditional Models*																	
gf	.35	<.001	.30,	.40		.17	<.001	.09,	.25	.34	<.001	.29,	.39	.16	<.001	.08,	.23
C	.02	.454	−.04,	.08		−.04	.138	−.11,	.03	.05	.013	−.00,	.11	−.02	.315	−.08,	.03
gf × C	-	-	-		-	-	-	-	-	.00	.983	−.06,	.06	.01	.683	−.08,	.11
Model fit	247.46 (23), *p* < .001, CFI = .930, RMSEA = .058, SRMR = .043									-							
AIC; aBIC	60,351.96; 60,438.59									74,561.66; 74,662.62							
R^2^	.235									.251							
*Conditional Models*																	
gf	.30	<.001	.46,	.70		.14	<.001	.11,	.40	.29	<.001	.30,	.44	.14	<.001	.08,	.25
C	−.01	.840	−.22,	.19		−.06	.044	−.40,	.05	.02	.242	−.04,	.09	−.04	.081	−.11,	.02
gf × C	-	-	-			-	-	-		.00	.987	−.07,	.07	.03	.460	−.09,	.14
School	−.16	<.001	−.21,	−.11		−.08	.001	−.15,	−.02	−.17	<.001	−.21,	−.12	−.08	<.001	−.13,	−.02
Female	.03	.119	−.02,	.08		−.06	.018	−.01,	.12	.03	.074	−.01,	.07	.05	.009	.00,	.10
HISEI	.19	<.001	.14,	−24		.16	<.001	.10,	.22	.21	<.001	−.16	.25	.14	<.001	.10,	.19
Model fit	359.96(38), *p* <.001, CFI = .916, RMSEA = .054, SRMR = .041									-							
AIC; aBIC	89,917.85; 90,063.18									113,184.09; 113,349.30							
R^2^	.270									.280							
*Note*. Standardized regression coefficients with exact *p*-values and 99% confidence intervals. Change = gains in competencies from grade 4 to grade 7; gf = fluid intelligence; C = conscientiousness; gf × C = interaction term between fluid intelligence and conscientiousness; School = non-academic track; HISEI= highest occupational prestige from both parents; AIC = Akaike information criterion (smaller values indicate better fit); aBIC = sample-size-adjusted Bayesian information criterion (smaller values indicate better fit).																	

**Table 4:** Latent change score models predicting reading competence baseline levels and gains in SC3.

	Model 1: Baseline									Model 2: Interaction							
	Competence T1					Change				Competence T1				Change			
Predictor	Est	*p*	99% CI			Est	*p*	99% CI		Est	*p*	99% CI		Est	*p*	99% CI	
*Unconditional Models*																	
gf	.49	<.001	.44,	.55		.20	<.001	.14,	.26	.50	<.001	.45,	.54	.20	<.001	.15,	.26
C	.09	<.001	.05,	.13		.02	.189	−.02,	.05	.09	<.001	.06,	.13	.02	.108	−.01,	.05
gf × C	-	-	-		-	-	-	-	-	.04	.014	-.00,	.09	.05	.002	.01,	.09
Model fit	27.73 (11) *p* = .004, CFI = .997, RMSEA = .018, SRMR = .016																
AIC; aBIC	79,428.85; 79,508.39									80,618.09; 80,707.94							
R^2^	.396									.397							
*Conditional Models*																	
gf	.36	<.001	.30,	.42		.16	<.001	.10,	.22	.36	<.001	.31,	.42	.16	<.001	.10,	.22
C	.00	.932	−.06,	.07		.01	.509	−.03,	.05	.01	.016	−.06,	.07	.01	.455	−.03,	.06
gf × C	-	-	-			-	-	-		.02	.022	−.04,	.08	.04	.036	−.01,	.10
School	−.24	<.001	−.30,	−.19		−.13	<.001	−.18,	−.08	−.24	<.001	−.28,	−.20	−.13	<.001	−.17,	−.09
Female	.10	<.001	.06,	.14		.05	<.001	.01,	.08	.10	<.001	.06,	.14	−.04	<.001	.01,	.08
HISEI	.11	<.001	.06,	.16		.09	<.001	.05,	.14	.11	<.001	.07,	.15	−.09	<.001	.05,	.13
Model fit	88.18(19), *p* < .001 CFI = .990, RMSEA = .027, SRMR = .019																
AIC; aBIC	120,585.84; 120,738.29									122,291.08; 122,450.82							
R^2^	.425									.425							
*Note*. Standardized regression coefficients with exact *p*-values and 99% confidence intervals. Change = gains in competencies from grade 7 to grade 9; gf = fluid intelligence; C = conscientiousness; gf × C = interaction term between fluid intelligence and conscientiousness; School = non-academic track; HISEI= highest occupational prestige from both parents; AIC = Akaike information criterion (smaller values indicate better fit); aBIC = sample-size-adjusted Bayesian information criterion (smaller values indicate better fit).																	

**Table 5:** Model comparisons of latent interaction models (B) with latent change models without the interaction term (A) in SC3.

Model	Log-Likelihood (L)	Scaling Correction Factor (scf)	Free Parameters (fp)	Δχ^2^	Δdf
*Reading*					
Latent Change Model (A)	–60,246.92	1.511	46		
Latent Interaction Model (B)	–61,097.54	0.986	48	38.37 *	2
*Note.* Δχ^2^ difference tests were computed based on the formula presented by Hildebrandt et al. (2009), that is, Δχ^2^ = −2 * (LB − LA)/c, where c = (scfB * fpB − scfA * fpA)/(fpB −– fpA). * χ^2^ difference test is statistically significant at *p* < .01.					

**Table 6:** Latent change score models predicting mathematic competence baseline levels and gains in SC2.

	Model 1: Baseline									Model 2: Interaction							
	Competence T1					Change				Competence T1				Change			
Predictor	Est	*p*	99% CI			Est	*p*	99% CI		Est	*p*	99% CI		Est	*p*	99% CI	
*Unconditional Models*																	
gf	.39	<.001	.33,	.44		.17	<.001	.07,	.26	.39	<.001	.34,	.44	.16	<.001	.08,	.25
C	.03	.238	−.03,	.09		.01	.854	−.07,	.08	.05	.023	−.01,	.10	.04	.118	−.03,	.10
gf × C	-	-	-		-	-	-	-	-	.05	.023	−.01,	.10	.04	.118	−.03,	.10
Model fit	299.67 (23), *p* < .001, CFI = .923, RMSEA = .064, SRMR = .050																
AIC; aBIC	59,286.57; 59,373.21									73,179.73; 73,280.69							
R^2^	.129									.140							
*Conditional Models*																	
gf	.35	<.001	.47,	.67		.18	<.001	.12,	.41	.34	<.001	.29,	.39	.18	<.001	.09,	.26
C	.02	.289	−.10,	.24		.02	.560	−.15,	.23	.04	.063	−.01,	.09	.05	.037	−.01,	.12
gf × C	-	-	-			-	-	-		.00	.865	−.06,	.07	.07	.053	−.02,	.16
School	−.16	<.001	−.21,	.11		−.09	.001	−.16,	−.02	−.16	<.001	−.20,	−.11	−.08	<.001	−.14	−.03
Female	−.11	<.001	−.16,	−.07		−.16	<.001	−.23,	−.09	−.11	<.001	−15,	−.07	−.15	<.001	−.21	−.10
HISEI	.21	<.001	.16,	.26		.11	<.001	.05,	.17	.23	<.001	.19,	.27	.13	<.001	.08	.18
Model fit	390.70 (38), *p* < .001, CFI = .917, RMSEA = .057, SRMR = .045																
AIC; aBIC	88,769.70; 88,915.03									111,680.56; 111,845.77							
R^2^	.176									.189							
*Note*. Standardized regression coefficients with exact *p*-values and 99% confidence intervals. Change = gains in competencies from grade 4 to grade 7; gf = fluid intelligence; C = conscientiousness; gf × C = interaction term between fluid intelligence and conscientiousness; School = non-academic track; HISEI= highest occupational prestige from both parents; AIC = Akaike information criterion (smaller values indicate better fit); aBIC = sample-size-adjusted Bayesian information criterion (smaller values indicate better fit).																	

**Table 7:** Latent change score models predicting mathematic competence baseline levels and gains in SC3.

	Model 1: Baseline									Model 2: Interaction							
	Competence T1					Change				Competence T1				Change			
Predictor	Est	*p*	99% CI			Est	*p*	99% CI		Est	*p*	99% CI		Est	*p*	99% CI	
*Unconditional Models*																	
gf	.65	<.001	.61,	.70		.27	<.001	.19,	.36	.65	<.001	.61,	.69	.28	<.001	.20,	.36
C	.05	.003	.01,	.09		.04	.003	.01,	.08	.04	.031	−.01,	.10	.06	.003	.01,	.11
gf × C	-	-	-		-	-	-	-	-	.05	.046	−.01	.11	.03	.340	−.05,	.10
Model fit	47.28 (11), *p* < .001, CFI = .995, RMSEA = .026, SRMR = .021																
AIC, aBIC	77,992.36, 78,071.90									79,188.01, 79,274.53							
R^2^	.196									.200							
*Conditional Models*																	
gf	.50	<.001	.45,	.70		.24	<.001	.16,	.32	.51	<.001	.46,	.55	.24	<.001	.16,	.32
C	−.01	.732	−.05,	.04		.04	.108	−.02,	.09	.01	.725	−.05,	.06	.04	.089	−.02,	.09
gf × C	-	-	-			-	-	-		.02	.407	−.04,	.08	.02	.565	−.06,	.09
School	−.25	<.001	−.30,	−.20		−.20	<.001	−.25,	−.14	−.25	<.001	−.29,	−.21	−.20	<.001	−.24,	−.15
Female	−.14	<.001	−.17,	−.01		−.08	<.001	−.12,	−.04	−.14	<.001	−.17,	−.10	−.08	<.001	−.12,	−.04
HISEI	.12	<.001	.07,	.16		.12	<.001	.07,	.16	.12	<.001	.08,	.16	.12	<.001	.07,	.16
Model fit	88.88 (19), *p* < .001, CFI = .992, RMSEA = .027, SRMR = .019																
AIC, aBIC	118,640.91, 118,793.36									120,340.04, 120,499.78							
R^2^	.246									.247							
*Note*. Standardized regression coefficients with exact *p*-values and 99% confidence intervals. Change = gains in competencies from grade 7 to grade 9; gf = fluid intelligence; C = conscientiousness; gf × C = interaction term between fluid intelligence and conscientiousness; School = non-academic track; HISEI= highest occupational prestige from both parents; AIC = Akaike information criterion (smaller values indicate better fit); aBIC = sample-size-adjusted Bayesian information criterion (smaller values indicate better fit).																	

## Data Availability

In the current study, we analyzed publicly available secondary data from the German National Educational Panel Study (NEPS) that can be downloaded as anonymized scientific use files from the NEPS website after concluding a data use agreement with the Leibniz Institute for Educational Trajectories (https://www.neps-data.de/Data-Center/Data-Access).

## Note

1 See Figure S1 in the online Supplementary Materials for the figure including confidence bands.

## Supplementary Materials

The following supporting information can be downloaded at: https://www.mdpi.com/article/10.3390/jintelligence10020027/s1, Figure S1: Interaction between fluid intelligence and conscientiousness in predicting reading competence development in SC3 (with confidence bands); Table S1: Means, standard deviations, and correlations of SC2 full sample; Table S2: Means, standard deviations, and correlations of SC3 full sample; Table S3: Latent Change Score Models Predicting Reading Competence Baseline Levels and Gains in SC2 full Sample; Table S4: Latent Change Score Models Predicting Reading Competence Baseline Levels and Gains in SC3 full Sample; Table S5: Latent Change Score Models Predicting Mathematic Competence Baseline Levels and Gains in SC2 full Sample; Table S6: Latent Change Score Models Predicting Mathematic Competence Baseline Levels and Gains in SC3 full Sample; Table S7: Model Comparisons of Latent Interaction Models (B) with Latent Change Models Without the Interaction Term (A) in SC3 full Sample.
