# Supplementary material for: Fluid Intelligence and Competence Development in Secondary Schooling: No Evidence for a Moderating Role of Conscientiousness
Source: J Intell. 2022 Apr 28;10(2):27. doi: 10.3390/jintelligence10020027 (PMC9149944; doi:10.3390/jintelligence10020027)
Supplement: Supplementary file 1 [file jintelligence-10-00027-s001.zip › jintelligence-1596103-supplementary.pdf]

ONLINE SUPPLEMENTARY MATERIAL

Fluid Intelligence and Competence Development in Secondary Schooling: No Evidence for a Moderating Role of Conscientiousness

Naemi D. Brandt & Clemens M. Lechner

As a robustness check, we estimated all models again using the full sample (participants that provided data at either T1 or T2). Resulting sample sizes were $N = 9{,}318$ in SC2 (average age of 9.31 at T1, 51% female, and 83% attending the academic track), and $N = 7{,}861$ in SC3 (average age of 12.53 at T1, 49% female, and 51% attending the academic track). The pattern of results remained highly stable across samples used except for very minor differences: In SC2, the effect of conscientiousness on reading and math competence at T1 reached statistical significance, although parameter estimates are highly similar and effect sizes remain very small. In SC3, the interaction of conscientiousness and fluid intelligence was related to competence levels in addition to predicting change in reading in the smaller sample (again with highly similar parameter estimates).

Table S1. *Means, standard deviations, and correlations of SC2 full sample.*

Variable	*M*	*SD*	1	2	3	4	5	6	7	8
1. Fluid intelligence	4.43	3.86								
2. Conscientiousness	3.26	0.52	.04*<br>[.02, .07]							
3. Female	0.51		.05*<br>[.02, .07]	.04*<br>[.02, .07]						
4. HISEI	58.95	19.68	.17*<br>[.15, .19]	.05*<br>[.02, .07]	-.01<br>[-.03, .01]					
5. Non-academic track	0.17		-.07*<br>[-.10, -.04]	-.13*<br>[-.17, -.10]	.01<br>[-.03, .04]	-.19*<br>[-.23, -.16]				
6. Reading WLE grade 4	-0.58	1.31	.18*<br>[.16, .20]	.12*<br>[.09, .14]	.08*<br>[.05, .10]	.33*<br>[.31, .35]	-.26*<br>[-.29, -.22]			
7. Reading WLE grade 7	0.18	1.30	.10*<br>[.06, .14]	.08*<br>[.04, .12]	.09*<br>[.05, .13]	.30*<br>[.26, .33]	-.24*<br>[-.28, -.20]	.57*<br>[.54, .59]		
8. Math WLE grade 4	4.62	1.15	.23*<br>[.20, .25]	.11*<br>[.09, .14]	-.06*<br>[-.08, -.03]	.34*<br>[.32, .37]	-.26*<br>[-.29, -.23]	.65*<br>[.64, .67]	.49*<br>[.46, .53]	
9. Math WLE grade 7	5.86	1.22	.17*<br>[.13, .20]	.15*<br>[.11, .19]	-.14*<br>[-.18, -.11]	.31*<br>[.28, .35]	-.27*<br>[-.31, -.23]	.52*<br>[.50, .55]	.58*<br>[.54, .62]	.65*<br>[.62, .67]

Note. *M* and *SD* are used to represent mean and standard deviation, respectively. For binary variables gender and school track, proportions are shown. Values in square brackets indicate the 95% confidence interval for each correlation. * indicates $p < .01$

Table S2. *Means, standard deviations, and correlations of SC3 full sample.*

Variable	*M*	*SD*	1	2	3	4	5	6	7	8
1. Fluid intelligence	4.55	3.90								
2. Conscientiousness	3.23	0.85	.01<br>[-.02, .03]							
3. Female	0.48		-.01<br>[-.03, .01]	.18*<br>[.15, .20]						
4. HISEI	56.02	20.09	.15*<br>[.12, .18]	.04<br>[.01, .07]	.00<br>[-.02, .03]					
5. Non-academic track	0.51		-.25*<br>[-.28, -.23]	-.04*<br>[-.07, -.01]	-.04*<br>[-.07, -.02]	-.40*<br>[-.42, -.37]				
6. Reading WLE grade 7	0.79	1.36	.14*<br>[.12, .17]	.04*<br>[.02, .07]	.10*<br>[.07, .12]	.31*<br>[.28, .34]	-.44*<br>[-.46, -.42]			
7. Reading WLE grade 9	1.33	1.12	.20*<br>[.17, .23]	.03<br>[.00, .06]	.10*<br>[.07, .13]	.33*<br>[.29, .36]	-.44*<br>[-.47, -.42]	.63*<br>[.62, .65]		
8. Math WLE grade7	0.82	1.23	.24*<br>[.22, .26]	-.01<br>[-.04, .01]	-.14*<br>[-.17, -.12]	.35*<br>[.33, .38]	-.50*<br>[-.52, -.48]	.60*<br>[.58, .62]	.52*<br>[.50, .54]	
9. Math WLE grade 9	1.60	1.19	.26*<br>[.24, .29]	.01<br>[-.02, .04]	-.12*<br>[-.15, -.09]	.37*<br>[.34, .40]	-.52*<br>[-.54, -.49]	.57*<br>[.55, .59]	.58*<br>[.56, .60]	.74*<br>[.72, .75]

Note. M and *SD* are used to represent mean and standard deviation, respectively. For binary variables gender and school track, proportions are shown. Values in square brackets indicate the 95% confidence interval for each correlation. * indicates $p < .01$.

Table S3. *Latent Change Score Models Predicting Reading Competence Baseline Levels and Gains in SC2 full Sample.*

j

	Model 1: Baseline								Model 2: Interaction							
	Competence T1				Change				Competence T1				Change			
Predictor	Est	*p*	99% CI		Est	*p*	99% CI		Est	*p*	99% CI		Est	*p*	99% CI	
Unconditional Models																
gf	.36	<.001	.33,	.40	.17	<.001	.09,	.26	.36	<.001	.33,	.40	.16	<.001	.08,	.23
C	.05	.002	.01,	.10	-.04	.151	-.11,	.03	.08	<.001	.04,	.12	-.02	.402	-.08,	.04
gf × C	-	-	-	-	-	-	-	-	-.00	.953	-.04,	.04	.03	.494	-.07,	.12
Model fit	347.62 (23), *p*<.001, CFI = .943, RMSEA = .051, SRMR =.040								-							
AIC, aBIC	111524.26, 111630.85								138645.74, 138770.00							
R^2	.222								.236							
Conditional Models																
gf	.29	<.001	.25,	.33	.15	<.001	.06,	.23	.28	<.001	.25,	.32	.13	<.001	.06,	.21
C	.02	.184	-.02,	.06	-.06	.036	-.13,	.01	.04	.004	.00,	.08	-.04	.072	-.10,	.02
gf × C	-	-	-		-	-	-		-.00	.827	-.04,	.04	.04	.305	-.06,	.13
School	-.15	<.001	-.20,	-.11	-.09	<.001	-.16,	-.03	-.16	<.001	-.21,	-.12	-.09	<.001	-.14,	-.03
Female	.05	<.001	.01,	.09	.07	.005	.01,	.13	.05	<.001	-.02,	.08	.06	.001	.01,	.11
HISEI	.24	<.001	.20,	.28	.17	<.001	.11,	.23	.26	<.001	.22	.29	.16	<.001	.11,	.21
Model fit	524.83(38), *p*<.001, CFI = .928, RMSEA = .048, SRMR =.038								-							
AIC, aBIC	163583.36, 163762.24								223369.33, 223580.61							
R^2	.249								.261							

Note. Standardized regression coefficients with exact *p*-values and 99% confidence intervals. Change = Gains in Competencies from Grade 4 to Grade 7. gf = fluid intelligence, C = conscientiousness. gf × C = interaction term between fluid intelligence and conscientiousness. School = non-academic track, HISEI= highest occupational prestige from both parents, AIC = Akaike Information Criterion (smaller values indicate better fit), aBIC = sample-size-adjusted Bayesian Information Criterion (smaller values indicate better fit).

Table S4. *Latent Change Score Models Predicting Reading Competence Baseline Levels and Gains in SC3 full Sample.*

	Model 1: Baseline								Model 2: Interaction							
	Competence T1				Change				Competence T1				Change			
Predictor	Est	*p*	99% CI		Est	*p*	99% CI		Est	*p*	99% CI		Est	*p*	99% CI	
Unconditional Models																
gf	.49	<.001	.44,	.54	.20	<.001	.14,	.27	.49	<.001	.45,	.54	.21	<.001	.16,	.27
C	.08	<.001	.05,	.12	.02	.200	-.02,	.05	.09	<.001	.06,	.13	.02	.174	-.02,	.05
gf × C	-	-	-	-	-	-	-	-	.05	.009	.00,	.09	.05	.003	.01,	.09
Model fit	28.12 (11) *p* = .004, CFI = .997, RMSEA = .016, SRMR = .015															
AIC, aBIC	97748.50, 97834.26								112014.49, 112115.85							
R^2	.395								.398							
Conditional Models																
gf	.36	<.001	.31,	.42	.16	<.001	.10,	.22	.37	<.001	.32,	.42	.16	<.001	.10,	.22
C	.01	.727	-.05,	.06	.01	.492	-.03,	.05	.02	.394	-.04,	.08	.01	.533	-.03,	.05
gf × C	-	-	-		-	-	-		.03	.216	-.03,	.08	.05	.033	-.01,	.10
School	-.24	<.001	-.28,	-.17	-.13	<.001	-.17,	-.08	-.22	<.001	-.26,	-.18	-.12	<.001	-.16,	-.08
Female	.10	<.001	.07,	.14	.05	<.001	.01,	.08	.10	<.001	.07,	.13	.04	.001	.01,	.08
HISEI	.12	<.001	.08,	.16	.10	<.001	.05,	.14	.13	<.001	.09,	.17	.10	<.001	.06,	.14
Model fit	93.95(19), *p* <.001 CFI = .990, RMSEA = .025, SRMR = .017															
AIC, aBIC	151348.75, 151513.86								176384.89, 176566.85							
R^2	.424								.426							

Note. Standardized regression coefficients with exact *p*-values and 99% confidence intervals. Change = Gains in Competencies from Grade 7 to Grade 9. gf = fluid intelligence, C = conscientiousness. gf × C = interaction term between fluid intelligence and conscientiousness. School = non-academic track, HISEI= highest occupational prestige from both parents, AIC = Akaike Information Criterion (smaller values indicate better fit), aBIC = sample-size-adjusted Bayesian Information Criterion (smaller values indicate better fit).

Table S5. *Latent Change Score Models Predicting Mathematic Competence Baseline Levels and Gains in SC2 full Sample.*

	Model 1: Baseline								Model 2: Interaction							
	Competence T1				Change				Competence T1				Change			
Predictor	Est	*p*	99% CI		Est	*p*	99% CI		Est	*p*	99% CI		Est	*p*	99% CI	
Unconditional Models																
gf	.40	<.001	.36,	.44	.17	<.001	.07,	.27	.40	<.001	.37,	.44	.17	<.001	.08,	.26
C	.03	.094	-.02,	.08	.00	.901	-.07,	.08	.05	.001	.01,	.09	.04	.127	-.03,	.12
gf × C	-	-	-	-	-	-	-	-	.01	.458	-.03,	.06	.08	.063	-.03,	.19
Model fit	474.71 (23), *p*<.001, CFI = .927, RMSEA =.060, SRMR = .050															
AIC, aBIC	109437.42, 109544.02								136180.03, 136304.32							
R^2	.135								.146							
Conditional Models																
gf	.34	<.001	.30,	.38	.19	<.001	.09,	.21	.34	<.001	.30,	.38	.19	<.001	.10,	.28
C	.02	.229	-.02,	.07	.02	.555	-.06,	.09	.04	.021	.00,	.07	.05	.052	-.02,	.11
gf × C	-	-	-		-	-	-		.01	.454	-.03,	.06	.08	.063	-.03,	.19
School	-.16	<.001	-.21,	.11	-.09	.001	-.16,	-.02	-.16	<.001	-.21,	-.12	-.08	<.001	-.14	-.02
Female	-.10	<.001	-.14,	-.07	-.16	<.001	-.22,	-.09	-.10	<.001	-.13,	-.07	-.15	<.001	-.20	-.11
HISEI	.25	<.001	.21,	.28	.12	<.001	.05,	.19	.26	<.001	.23,	.29	.14	<.001	.09	.20
Model fit	609.01 (38), *p* <.001, CFI = .921, RMSEA =.052, SRMR=.045															
AIC, aBIC	161416.02, 161594.91								220802.16, 221013.45							
R^2	.182								.194							

Note. Standardized regression coefficients with exact *p*-values and 99% confidence intervals. Change = Gains in Competencies from Grade 4 to Grade 7. gf = fluid intelligence, C = conscientiousness. gf × C = interaction term between fluid intelligence and conscientiousness. School = non-academic track, HISEI= highest occupational prestige from both parents, AIC = Akaike Information Criterion (smaller values indicate better fit), aBIC = sample-size-adjusted Bayesian Information Criterion (smaller values indicate better fit).

Table S6. *Latent Change Score Models Predicting Mathematic Competence Baseline Levels and Gains in SC3 full Sample.*

	Model 1: Baseline								Model 2: Interaction							
	Competence T1				Change				Competence T1				Change			
Predictor	Est	*p*	99% CI		Est	*p*	99% CI		Est	*p*	99% CI		Est	*p*	99% CI	
Unconditional Models																
gf	.65	<.001	.61,	.69	.28	<.001	.19,	.34	.66	<.001	.62,	.69	.29	<.001	.21,	.37
C	.04	.005	.00,	.08	.04	.004	.01,	.08	.04	.025	-.01,	.09	.06	.002	.01,	.11
gf × C	-	-	-	-	-	-	-	-	.05	.010	.00	.11	.03	.355	-.05,	.10
Model fit	51.04 (11), *p*<.001, CFI = .995, RMSEA = .024, SRMR = .020															
AIC, aBIC	96472.86, 96558.75								110752.59, 110850.33							
R^2	.198								.203							
Conditional Models																
gf	.51	<.001	.46,	.55	.24	<.001	.16,	.32	.51	<.001	.47,	.55	.24	<.001	.17,	.33
C	.01	.696	-.04,	.06	.03	.117	-.02,	.09	.01	.471	-.03,	.06	.04	.069	-.02,	.09
gf × C	-	-	-		-	-	-		.03	.125	-.02,	.09	.02	.608	-.06,	.09
School	-.24	<.001	-.29,	-.19	-.20	<.001	-.25,	-.14	-.23	<.001	-.27,	-.19	-.20	<.001	-.24,	-.15
Female	-.13	<.001	-.16,	-.10	-.07	<.001	-.12,	-.04	-.13	<.001	-.16,	-.10	-.08	<.001	-.11,	-.04
HISEI	.12	<.001	.08,	.16	.11	<.001	.07,	.16	.12	<.001	.09,	.16	.12	<.001	.08,	.16
Model fit	93.07 (19), *p*<.001, CFI = .993, RMSEA=.025, SRMR =.017															
AIC, aBIC	149399.74, 149564.85								174435.79, 174617.74							
R^2	.252								.254							

Note. Standardized regression coefficients with exact *p*-values and 99% confidence intervals. Change = Gains in Competencies from Grade 7 to Grade 9. gf = fluid intelligence, C = conscientiousness. gf × C = interaction term between fluid intelligence and conscientiousness. School = non-academic track, HISEI= highest occupational prestige from both parents, AIC = Akaike Information Criterion (smaller values indicate better fit), aBIC = sample-size-adjusted Bayesian Information Criterion (smaller values indicate better fit).

Table S7. *Model Comparisons of Latent Interaction Models (B) with Latent Change Models Without the Interaction Term (A) in SC3 full Sample.*

Model	loglikelihood (L)	Scaling correction factor (scf)	Free parameters (fp)	$\Delta\chi^2$	Δdf
Reading					
Latent Change Model (A)	–39676.18	1.256	24		
Latent Interaction Model (B)	–40282.05	0.977	27	106.69*	3

Note. $\Delta\chi^2$ differences tests were computed based on the formula presented by Hildebrandt et al. (2009), that is $\Delta\chi^2$ = -2 * (LB - LA) / c; where c = (scfB * fpB – scfA * fpA) / (fpB – fpA). *χ^2 difference test is statistically significant at $p < .01$.

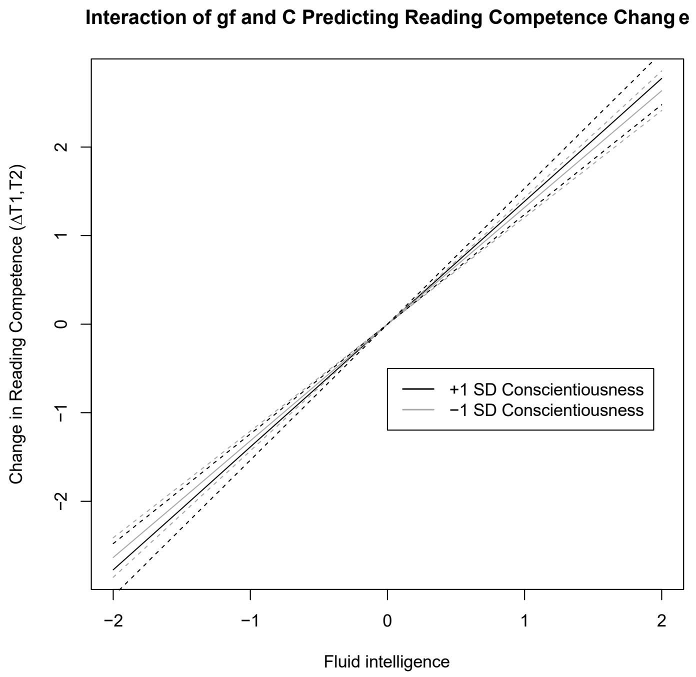

Figure S1. *Note.* Interaction between fluid intelligence and conscientiousness in predicting reading competence development in SC3. The dark gray line represents the association between fluid intelligence and change in reading competence for high levels of conscientiousness (+1 SD above the sample mean), whereas the light gray line represents the same association for low levels of conscientiousness (–1 SD below the sample mean). Dotted lines represent 95% confidence bands (in dark grey for high levels of conscientiousness and in light gray for low levels of conscientiousness).
